# Cigarette smoking and cardio-renal events in patients with atherosclerotic renal artery stenosis

**DOI:** 10.1371/journal.pone.0173562

**Published:** 2017-03-17

**Authors:** Christopher A. Drummond, Pamela S. Brewster, Wencan He, Kaili Ren, Yanmei Xie, Katherine R. Tuttle, Steven T. Haller, Kenneth Jamerson, Lance D. Dworkin, Donald E. Cutlip, Timothy P. Murphy, Ralph B. D’Agostino, William L. Henrich, Jiang Tian, Joseph I. Shapiro, Christopher J. Cooper

**Affiliations:** 1 Department of Medicine, University of Toledo College of Medicine and Life Sciences, Toledo, OH, United States of America; 2 Department of Mathematics, University of Toledo, Toledo, OH, United States of America; 3 Division of Nephrology, University of Washington School of Medicine, Providence Health Care, Spokane, WA, United States of America; 4 Department of Medicine, University of Michigan, Ann Arbor, MI, United States of America; 5 Harvard Clinical Research Institute, Boston, MA, United States of America; 6 Departments of Medicine and Diagnostic Imaging, Rhode Island Hospital and Alpert Medical School of Brown University, Providence, RI, United States of America; 7 University of Texas Health Science Center, San Antonio TX, United States of America; 8 Joan C. Edwards School of Medicine, Marshall University, Huntington, WV, United States of America; University Jean MONNET of SAINT-ETIENNE, UNITED STATES

## Abstract

Cigarette smoking causes cardiovascular disease and is associated with poor kidney function in individuals with diabetes mellitus and primary kidney diseases. However, the association of smoking on patients with atherosclerotic renal artery stenosis has not been studied. The current study utilized data from the Cardiovascular Outcomes in Renal Atherosclerotic Lesions (CORAL, NCT00081731) clinical trial to evaluate the effects of smoking on the risk of cardio-renal events and kidney function in this population. Baseline data showed that smokers (n = 277 out of 931) were significantly younger at enrollment than non-smokers (63.3±9.1 years vs 72.4±7.8 years; p<0.001). In addition, patients who smoke were also more likely to have bilateral renal artery stenoses and peripheral vascular disease (PVD). Longitudinal analysis showed that smokers experienced composite endpoint events (defined as first occurrence of: stroke; cardiovascular or renal death; myocardial infarction; hospitalization for congestive heart failure; permanent renal replacement; and progressive renal insufficiency defined as 30% reduction of GFR from baseline sustained for ≥ 60 days) at a substantially younger age compared to non-smokers (67.1±9.0 versus 76.1±7.9, p<0.001). Using linear regression and generalized linear modeling analysis controlled by age, sex, and ethnicity, smokers had significantly higher cystatin C levels (1.3±0.7 vs 1.2±0.9, p<0.01) whereas creatinine and estimated glomerular filtration rate (eGFR) were not different from non-smokers. From these data we conclude that smoking has a significant association with deleterious cardio-renal outcomes in patients with renovascular hypertension.

## Introduction

Despite tobacco control programs aimed at reducing consumption, cigarette smoking still causes 443,000 deaths each year in the United States according to the Centers for Disease Control and Prevention [1, 2]. Cigarette smoking is widely known to have adverse health effects on cardiovascular disease, chronic obstructive lung disease, liver disease and cancer [3–5], and may have adverse effects on kidney function [6]. The deleterious effects of smoking on kidney function have been reported in patients with diabetes mellitus and primary kidney diseases including polycystic kidney disease, glomerulonephritis and lupus nephritis[3–9]. Recently, epidemiologic studies demonstrated that smoking increases the risk of chronic kidney disease progression and kidney failure in patients with diabetes and hypertension [10, 11]. However, the importance of active smoking in patients with renal artery stenosis has not been described.

Renal artery stenosis (RAS) is a common problem and is present in roughly 1–5% of the 60 million Americans with hypertension [12–14]. This is considered a disease of older persons with most studies having a mean age for RAS greater than 70 years [15]. Low kidney function is common and there is a strikingly high rate of adverse cardiovascular events and mortality [16]. Smoking is a known risk factor for atherosclerosis, and a smoking history is typical in patients with renal artery stenosis [17]. However, the association between smoking and cardio-renal outcomes has not been studied in patients with atherosclerotic renal disease. This study used data from the Cardiovascular Outcomes in Renal Atherosclerotic Lesions (CORAL, NCT00081731) [18] clinical trial to evaluate changes in cardio-renal outcomes and secondary parameters in RAS patients who are active smokers.

## Materials and methods

### Study population

CORAL is a prospective, international, multicenter clinical trial that randomly assigned 931 participants with atherosclerotic renal artery stenosis who received optimal medical therapy to stenting versus no stenting from May 2005 through January 2010. The detailed description and analysis of the endpoints have previously been described [18]. For the current study the primary outcome was the composite endpoint defined as the first occurrence of any secondary endpoints (cardiovascular or renal death; myocardial infarction (ST segment elevation or silent MI); stroke; congestive heart failure (CHF); progressive renal insufficiency (≥30% decline in GFR over the period of 6 months [19]); and permanent renal replacement. Active cigarette smoking was defined as tobacco use within the year prior to enrollment in the study. The study was approved by the Institutional Review Board at the University of Toledo (IRB104827). All of the centers followed the institutional and study guidelines. All participating patients have provided written informed consent. Detailed study entry criteria have been published [18]. Patients with renal artery stenosis of at least 60% were eligible if they had hypertension while receiving two or more antihypertensive agents or had an estimated glomerular filtration rate (eGFR) less than 60 ml/minute/1.73m^2^ [18]. Angiograms were analyzed for verification of stenosis by the Angiography Core Lab for the study at the University of Virginia [18].

### Statistical analysis

Study data are presented as continuous (mean ± Standard Deviation [SD]), categorical (number and percentage), and medians (with interquartile range [IQR]). Comparisons of continuous data were evaluated with ANOVA and two-sample *t* tests, while for categorical variables, the Fisher exact test was used and odds ratios (OR) were calculated. Statistical significance was defined as a p-value <0.05. Data was tested for normal distribution using the Shapiro-Wilk test and nonparametric Mann-Whitney U rank test was used if the assumption of normality was violated. Where possible, the data was log-transformed to approximate a normal distribution and parametric analysis was undertaken. All analyses were performed using R software (version 3.0.0) and SAS (version 9.3).

### Multiple variable models and longitudinal analyses

Univariate analysis was performed comparing smokers to non-smokers on baseline characteristics, and variables with p<0.05 were included in multiple variable analyses using multiple linear regression model and generalized linear model (GLM) with application of stepwise model selection. Each of the baseline factors were analyzed as response variables adjusted for age and smoking status to correct for potential age bias and to guide in the selection of factors for the multivariable models. All multiple variable analyses were controlled for age, sex, ethnicity (Hispanic/Latino) and body mass index (BMI, weight in kilograms divided by the square of the height in meters). Longitudinal analyses using linear mixed effect model were performed using time (continuous) as the random coefficient to assess the effects of smoking on measures of kidney function (eGFR, cystatin C, serum creatinine concentrations) and damage (urine albumin-to-creatinine ratio [UACR]) up to 5 years. The models were checked using two versions of the mixed effect model: one version contained both time as a random coefficient effect and fixed effects, while the other version considered time as a fixed event. ANOVA was used to compare the residual sums of squares for the 2 models. The random effect model was selected as it yielded lower AIC and BIC measures.

### Event analysis

Due to the age discrepancy between smokers and non-smokers, we selected age-at-event as the time-scale for hazard risk since the covariate of interest (smoking) is not independent of age and the Cox model using time-to-event may incur bias even when adjusting for age [20–23]. Moreover, smokers accrue risk over their lifetime that would not be reflected if time-to-events were used as the response. Age-at-event outcomes were expressed using Kaplan-Meier estimates with comparisons between smoking status groups using the log-rank statistic. Hazard risk ratios were calculated using the Cox proportional-hazards model, and the comparison between smoking groups was evaluated using both survival time and age-at-event as the response variables. Model diagnostics were performed using the Cox-Snell residual plot test to check for goodness of fit. The proportional hazards assumption tests from the R function cox.zph in the survival package for all the survival models had p-values >0.05 indicating that the null hypothesis of proportional hazards was not rejected. The extended Cox model was used to test for interaction among model predictors and time. The time-dependent covariates were generated by building interactions of the predictors and a function of survival age (age-to-event), and were included in the models. The p-values for all time-covariate interactions in all of the survival models were greater than 0.05, confirming the null hypothesis assumption of proportional hazards, and indicating that the fitted Cox regression hazard models are adequate. The predicted probability of the binary occurrence of the composite endpoint or any of the secondary endpoint events was calculated using receiver operating characteristic (ROC) for logistic regression with adjustment for age, sex, ethnicity and BMI.

## Results

### Baseline characteristics comparison of smokers and non-smokers

**Fig 1:**
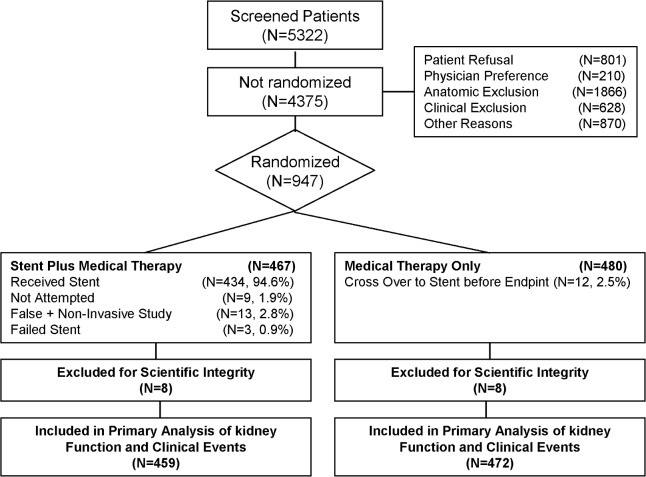
CONSORT flow diagram of CORAL study.

**Fig 2:**
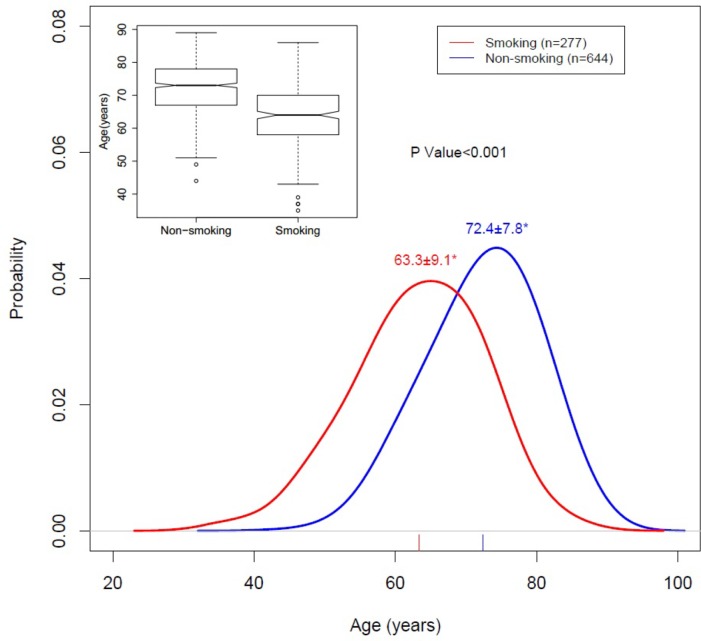
Age in years for smokers and non-smokers at study enrollment. The Red curve represents the distribution of age for smokers (N = 277) and the blue curve represents age at study enrollment for non-smokers (N = 644). Numbers at the peak of the distribution curves are the mean years of age at study enrollment ± SD for the patient populations as defined above. The insert boxplot shows age with interquartile ranges and 95% confidence intervals differentiated by smoking status. The asterisk (*) indicates that the means are significantly different (p<0.001) as determined by two-sample t-test.

The CONSORT flow diagram of CORAL study is shown in Fig 1. There were 277 (30%) smokers among the 931 participants in the CORAL trial. The median follow-up period was 43 months (interquartile range of 31 to 55). Smokers were younger than non-smoking participants at enrollment (63.3 ± 9.1 years old versus 72.4 ± 7.8 years old, p<0.001; Fig 2). Other baseline characteristics shown in Table 1 demonstrate no difference in renal artery stenosis severity between groups based on degree of stenosis (68.8±11.3% versus 68.5±11.7%, p = 0.68. Participants who smoke tended to be taller (66.4±4.2 inches versus 65.7±3.9 inches, p<0.001), leaner (172.3±37.1 lbs versus 176.9±36.2 lbs, p = 0.087) and had a significantly lower BMI (28.3±5.6 versus 29.7±5.6, p<0.001). Smokers were more likely to be on anti-platelet medication (75% versus 65%, p = 0.012), and were on fewer hypertensive drugs (1.8±1.5 versus 2.1±1.5, p = 0.015). In generalized linear models controlling for age, sex, ethnicity and BMI (Table 2), it was found that smokers were more likely to have peripheral vascular disease (p<0.001) and bilateral renal artery stenosis (p<0.05). The rate of diabetes amongst smokers was significantly lower than in non-smokers (p<0.001) which may be attributable to their younger age and leaner weight.

**Table 1:** Baseline clinical characteristics comparisons by smoking status within the last year.

Baseline Characteristics*	Non-smoking (n = 654)	Smoking (n = 277)	P-value
**Demographic/physical examination**			
**Age (yr)**	**72.4±7.8**	**63.3±9.1**	**<0.001**
Male sex	317(49)	133(48)	0.77
White race	584(91)	255(92)	0.53
Hispanic/Latino	39(6)	15(5)	0.76
United States as country origin	534(83)	233(84)	0.70
**Height (in)**	**65.7±3.9**	**66.4±4.2**	**<0.01**
Weight (lb)	176.9±36.2	172.3±37.1	0.087
**BMI (kg/m**^**2**^**)**	**29.7±5.6**	**28.3±-5.**	**<0.001**
Systolic BP (mmHg)	150.8±22.7	148.4±24.2	0.15
**Diastolic BP (mmHg)**	**77.9±12.9**	**80.1±13.6**	**0.021**
Systolic BP at goal	158(25)	83(30)	0.10
**Laboratory values (assessed by core lab)**			
Creatinine (mg/dl)	1.3±0.5	1.2±0.5	0.10
Cystatin C (mg/L)	1.3±0.5	1.3±0.5	0.95
**MDRD-eGFR (ml/min per 1.73 m**^**2**^**)**	**59.6±22.4**	**65.7±27.2**	**0.002**
**CKD-EPI creatinine formula**	**56.1±20.8**	**63.3±24.5**	**<0.001**
CKD-EPI cystatin C formula	59.9±24.1	62.1±24.9	0.23
**CKD-EPI Creatinine- cystatin C formula**	**58.1±21.7**	**62.8±25.8**	**<0.01**
Potassium (mmol/L)	4.2±0.6	4.2±0.5	0.33
Urine albumin creatinine ratio (μg/mg)	217.1±700.7	196.3±717.0	0.7
Dipstick proteinuria ≥ 100 mg/dl	32(5)	12(4)	0.74
**Angiographic findings**			
% stenosis, assessed by core laboratory	68.5±11.7	68.8±11.3	0.68
% stenosis, assessed visually at site	75.7±10.9	76.7±10.5	0.24
**Risk factors/indications**			
**Creatinine ≥ 1.6 (mg/dl)**	**464(72)**	**220(80)**	**0.017**
**Peripheral vascular disease**	**280(44)**	**180(66)**	**<0.001**
Hyperlipidemia	579(90)	236(88)	0.24
Prior myocardial infarction	173(27)	86(31)	0.26
Prior transient ischemic accident	127(20)	54(20)	0.99
Angina	69(12)	34(14)	0.49
Cardiovascular disease	361(61)	164(65)	0.44
**Diabetes mellitus**	**240(38)**	**66(24)**	**<0.001**
History of heart failure	91(14)	31(11)	0.24
**Chronic kidney disease**	**424(66)**	**148(53)**	**<0.001**
**CKD Stage**			
**Mild**	**239(37)**	**95(35)**	**0.0021**
**Moderate**	**288(45)**	**107(39)**	**<0.001**
**Severe**	**38(6)**	**13(5)**	**0.028**
Bilateral disease**	113(22)	58(26)	0.3
**Medication Use**			
Renin-angiotensin inhibitors	298(49)	118(49)	0.88
Diuretic	250(42)	91(37)	0.19
Aldosterone antagonist	17(3)	9(3)	0.66
*β*-Blocker	319(54)	123(49)	0.18
*α*-Blocker	102(17)	35(13)	0.19
*αβ*-Blocker	62(10)	31(12)	0.47
Calcium-channel blocker	239(43)	84(36)	0.057
Renin inhibitor	6(1)	0(0)	0.19
Vasodilator	33(5)	10(4)	0.40
Nitrate	115(19)	55(22)	0.40
**Antiplatelet agent**	**361(65)**	**173(75)**	**0.012**
Lipid-lowering agent	352(68)	138(61)	0.093
**Total hypertensive medications**	**2.1±1.5**	**1.8±1.5**	**0.015**
Total all medications	3.4±2.1	3.2±2	0.13

*Data are expressed as the mean±SD or number (percentage). Comparisons were evaluated using two sample t-test for continuous data or Fisher’s exact test with odds ratio for categorical data.

**Bilateral disease was defined as stenosis of 60% or more of the diameter of at least one artery supplying each kidney.

Abbreviations: CI, confidence interval; yr, year; in, inch; lb, pound, BMI, body mass index (weight in kilograms divided by the square of the height in meters); BP, blood pressure; MDRD-eGFR, Modification of Diet in Renal Disease-estimated glomerular filtration rate; CKD-EPI, Chronic Kidney Disease Epidemiology Collaboration; and CKD, Chronic kidney disease.

**Table 2:** Multivariate linear regression and generalized linear regression comparing smokers and non-smokers on baseline characteristics as the response adjusted for age, sex and ethnicity.

Baseline Characteristics	Smoking: Yes Coefficient (p-value)
**Demographic/physical examination**	
Height (in)	0.74 (<0.002)
Weight (lb)	-14.0 (<0.001)
BMI	-3.00 (<0.001)
Systolic BP (mmHg)	0.43 (0.82)
Diastolic BP (mmHg)	-0.22 (0.83)
**Angiographic findings**	
% stenosis, assessed by core laboratory	1.1 (0.30)
% stenosis, assessed visually	2.0 (<0.05)
Laboratory values (assessed by core lab)**	
Creatinine (mg/dl)	0.014 (0.71)
MDRD-eGFR (ml/min per 1.73 m^2^)	-0.67 (0.72)
Cystatin C (mg/L)	0.10 (<0.01)
Potassium (mmol/L)	0.015 (0.21)
UACR (ug/mg)	-0.10 (0.50)
**Risk factors/indications**	
Creatinine ≥ 1.6 (mg/dl)	0.15 (0.43)
Peripheral vascular disease	1.04 (<0.001)
Hyperlipidemia	-0.19 (0.47)
Prior myocardial infarction	0.14 (0.42)
Prior transient ischemic accident	0.19 (0.35)
Angina	0.25 (0.33)
Cardiovascular disease	0.07 (0.70)
Diabetes	-0.85 (<0.001)
History of heart failure	-0.34 (0.17)
Chronic kidney disease	-0.002 (0.99)
Bilateral disease***	0.45 (<0.05)

Linear model was used for continuous data and generalized linear model (GLM) for categorical factors.

** Laboratory values were log transformed when statistical analysis was performed.

*** Bilateral disease was defined as stenosis of 60% or more of the diameter of at least one artery supplying each kidney.

Abbreviations: **in**: inch; **lb:** pound, **BMI:** body mass index (weight in kilograms divided by the square of the height in meters); **BP**: blood pressure; **MDRD-eGFR**: Modification of Diet in Renal Disease-estimated glomerular filtration rate; **UACR**: urine albumin creatinine ratio (measured as the log UACR).

### Smoking as a risk factor for cardio-renal events in patients with renal artery stenosis

**Fig 3:**
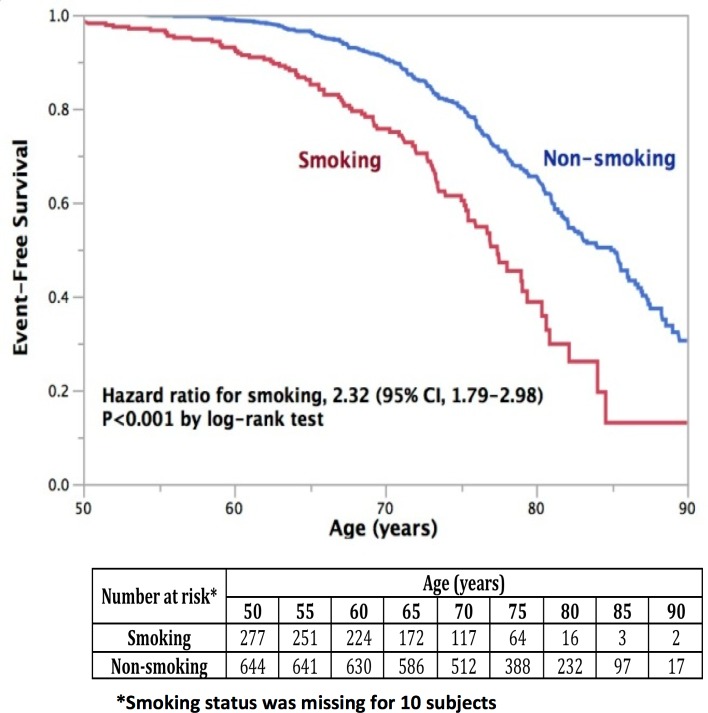
Kaplan-Meier curves of event-free survival for age-at-composite endpoint delineated by smoking status. The hazard ratio, assessed by log-rank test for age-at-composite endpoint delineated by smoking status, was 2.32 [1.79, 2.98], p<0.001.

In order to determine whether smoking is associated with the composite endpoint in these patients, event-free survival was plotted against age (Fig 3). Subjects were followed over a median follow-up of 43 months (IQR, 31 to 55). Smokers experienced these clinical events at a significantly younger age than non-smokers. Using age-at-event to determine Cox-proportional hazard ratios, participants who smoke were 2.32 (1.79, 2.98; p<0.001) times more likely to have a primary composite endpoint event at a younger age (67.1±9.0 vs 76.1±7.9, p<0.001) (Table 3 and Fig 3). Additionally, smokers were more likely to experience myocardial infarction (1.82 [1.06, 3.13]; p = 0.03) (68±9.1 vs 77±7.8, p<0.001), stroke (2.59 [1.27, 5.27]; p = 0.01) (68.9±9.1 vs 77.1±7.8, p<0.001), hospitalization for congestive heart failure (2.03 [1.17, 3.52]; p = 0.01) (68±9.0 vs 77±7.8, p<0.001), progressive renal insufficiency (2.38 [1.68, 3.38]; p<0.001) (67.5±9.0 vs 76.4±7.9, p<0.001), and death due to cardiovascular or renal disease (1.77 [1.02, 3.05]; p = 0.04) (68.1±9.1 vs 77.1±7.8, p<0.001). The only secondary clinical end-point observed not to be significantly affected by smoking was permanent renal replacement therapy (2.11 [0.82, 5.45]; p = 0.12) (68±9.0 vs 77±7.8, p<0.001), but the rate of this event was very low.

**Table 3:** Clinical events comparing smokers and non-smokers adjusted for sex, ethnicity and BMI using age-at-event (years) as the response.

Clinical Endpoint*	Smoking: Yes	Confidence Interval	P-Value
**Primary composite endpoint**	2.32	(1.79, 2.98)	<0.001
Secondary clinical endpoints**			
Cardiovascular or renal death	1.77	(1.02, 3.05)	0.04
Myocardial infarction	1.82	(1.06, 3.13)	0.03
Stroke	2.59	(1.27, 5.27)	0.01
Hospitalization for congestive heart failure	2.03	(1.17, 3.52)	0.01
Progressive renal insufficiency	2.38	(1.68, 3.38)	<0.001
Permanent renal replacement therapy	2.11	(0.82, 5.45)	0.12

* Rows display the hazard ratio, 95% confidence interval and p-value calculated using multivariable Cox proportional-hazards regression including DBP, peripheral vascular disease, and antihypertensive treatment. The multivariable, adjusted factors, and interaction term in the model were not significant except for smoking which is reported.

**Each component of the primary endpoint is included for the occurrence of the event.

### Longitudinal analysis of smoking effect on renal and cardiovascular function

**Fig 4:**
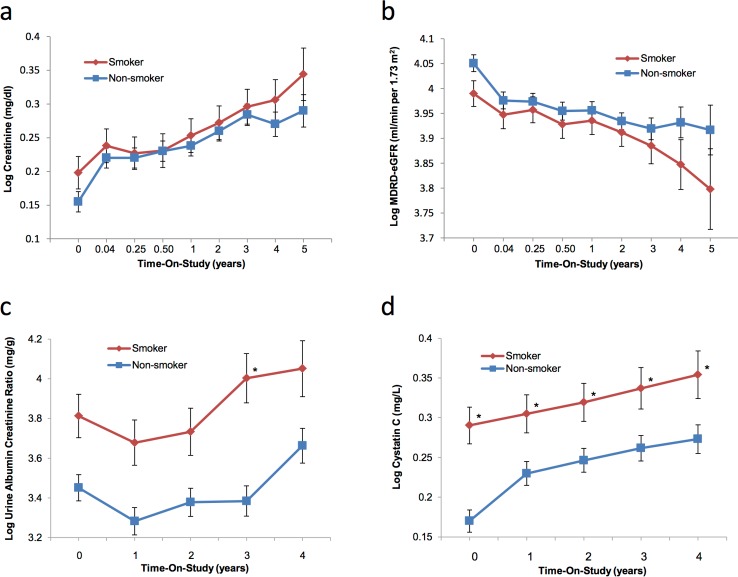
Longitudinal analysis of the effect of smoking on kidney function. Least square means measured over time-in-study are delineated by smoking status, and the panels display the following: a): log values of the means for creatinine (mg/dL); b): log values of the means for MDRD-eGFR (mL/min per 1.73m^2^); c): log values of the means for Urine Albumin to Creatinine Ratio (mg/g); and d): log values of the means for Cystatin C (mg/L). An asterisk (*) indicates the mean for smokers is significantly different than non-smokers value at same time point (p<0.05).

After adjusting for age, sex, diabetes, and BMI, there was no difference observed in measures of kidney function including serum creatinine and eGFR (Modified Diet in Renal Disease creatinine-based formula [MDRD-eGFR], Fig 4A and 4B) between smokers and non-smokers. Urine Albumin-to-Creatinine Ratio (UACR) increased more in smokers than in non-smokers over time and was significant at 3 years post-enrollment (Fig 4C, p<0.05). Cystatin C was also significantly higher in patients who smoked at baseline and throughout the study period (Fig 4D, p<0.05). Similar results were observed in analysis using the Chronic Kidney Disease Epidemiologic Collaboration (CKD-EPI) formulae for creatinine, cystatin C, and GFR combining creatinine and cystatin C (data not shown).

**Fig 5:**
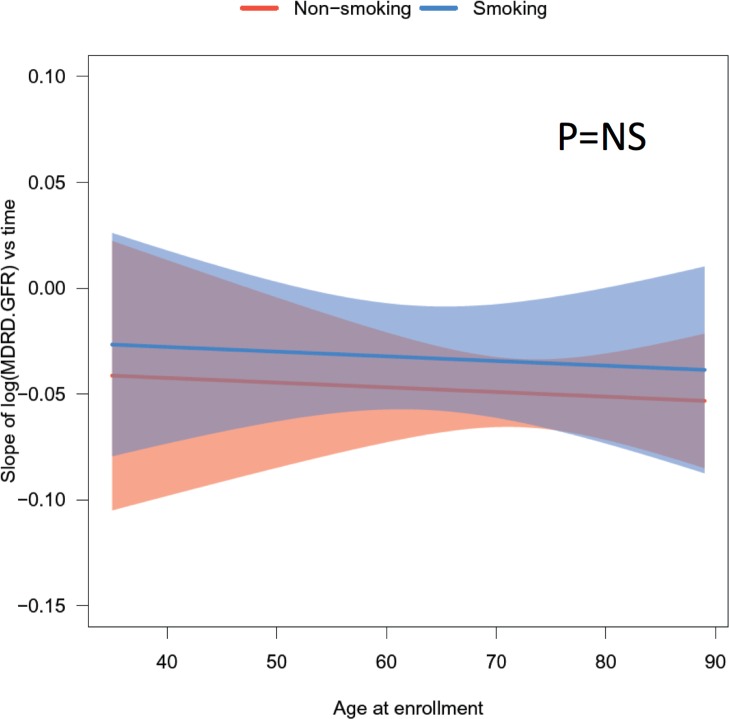
Fitted longitudinal slope of the natural log of MDRD-GFR over time-in-study by age at enrollment. For each individual, the longitudinal slope of MDRD-GFR was obtained by linear regression between the log of MDRD-GFR and time-in-study for that patient. These slopes were used as the response variable and fitted into a multiple variable regression model with age, sex, ethnicity, smoking, diabetes and BMI as covariates. The plot of the predicted slope of MDRD-GFR over time with 95% confidence intervals was generated for age at enrollment grouped by smoking status (red for smokers and blue for non-smokers).

**Fig 6:**
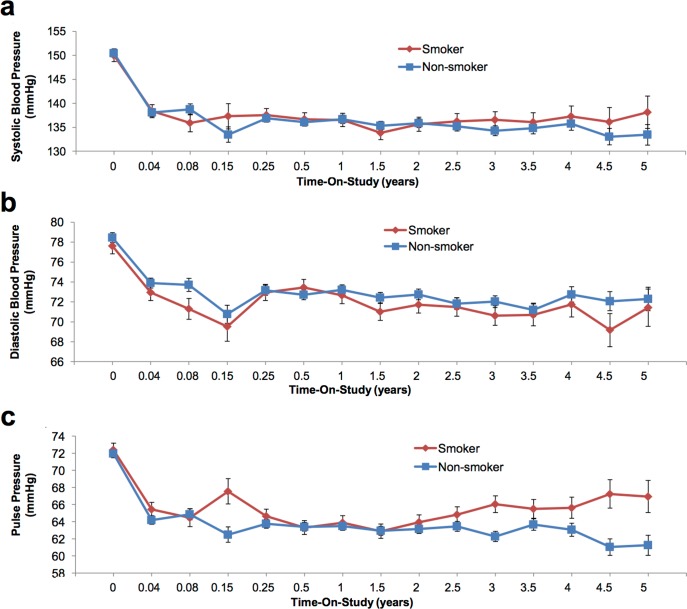
Longitudinal effect on blood pressure for smokers versus non-smokers. Graphs represent the following: a): systolic blood pressure (mmHg); b): diastolic blood pressure (mmHg); and c): pulse pressure (mmHg). Mean ± SD for the patient data from baseline through follow-up are given. No significant differences were observed.

To further examine the longitudinal effect of smoking on kidney function, the slope of natural log of eGFR versus time for each individual was obtained by linear regression to account for variable responses among subjects. These slopes were then used as the response variable and fitted into a multivariate regression model with age, sex, ethnicity, smoking, diabetes and BMI as covariates. The plot of predicted slope with 95% confidence intervals versus age at enrollment grouped by smoking status is shown in Fig 5, which showed a trend of decreased renal function in non-smokers that may be related to their older age, but it failed to reach statistical significance. In addition, hypertension measured by systolic or diastolic blood pressure, and pulse pressure were not significantly different between smokers and non-smokers (Fig 6).

## Discussion

We studied the effects of smoking on cardio-renal outcomes in patients with clinically significant renal artery stenosis and renovascular hypertension. The significant finding is that active smokers presented with renal artery stenosis at a much younger age and experienced cardio-renal events at a younger age than non-smoking participants. Smoking was significantly associated with cardiovascular or kidney death, myocardial infarction, stroke, hospitalization for congestive heart failure and progressive renal insufficiency. Even though smokers were 9.1 years younger at enrollment, they did not differ from non-smokers in renal artery stenosis severity. This is consistent with the known effect of smoking to accelerate atherosclerosis onset and progression [24–26], and that smoking increases risk for renal artery stenosis [17].

The effect of active smoking on renal function was less clear in the current study. We found no significant difference in renal function over time as evaluated by creatinine or creatinine-based eGFR among smokers and non-smokers, however, cystatin C levels were significantly higher in smokers at baseline and throughout follow up. The reason for this divergent finding on renal function estimation is not clear. The creatinine- or cystatin C-based eGFR may not be reliable indicators of renal function in populations who smoke as reported by Yamada [27]. Other factors beyond renal function can influence serum cystatin C levels [28]. Since cystatin C could be associated with inflammation [29], the observed increase of cystatin C in smokers could be due to the smoking induced inflammation. In addition, plasma creatinine level is dependent of tissue mass and BMI. The age as well as BMI in the smokers in this study is significantly different from the non-smokers. Therefore, we adjusted the age and BMI in our regression analysis in Table 2. It is also not clear whether smoking has any direct effect on plasma creatinine levels. It would prudent to consider these effects when assessing renal functions in smoking populations.

Other studies have found a relationship between smoking and kidney diseases including IgA glomerulonephritis or autosomal dominant polycystic kidney disease [3]. In the Multiple Risk Factor Intervention Trial (MRFIT) smoking was associated with an increased risk for ESRD and chronic kidney disease (CKD) [30]. This is supported by several other population-based longitudinal studies from countries including the United States, Japan, and Australia with enrollments ranging between 11,247 to over 100,000 individuals [6, 9, 31–34]. The PREVEND trial found a correlation between urine albumin excretion, a marker of kidney damage, and cigarette smoking [4–6, 8, 9, 35]. Yoon et al. found that smokers from the general population with a GFR ≥ 50 mL/min had less deterioration of kidney function but a higher risk of proteinuria, while in those with reduced GFR (< 50 mL/min) smoking was associated with a decline in GFR [36].

In the current study we only collected information on active smoking status and thus were unable to distinguish former smokers from participants who have never smoked, and did not have information on pack years and daily usage at baseline or through follow-up. This is a common issue for studies of this type [36–41]. While more detailed data on daily smoking consumption and cumulative pack years may assist to further refine the outcomes of this study, the overall findings remain important given the marked differences in outcomes between smokers and non-smokers.

In summary, the current study demonstrated that active smoking results in clinical significant renal artery stenosis, and adverse cardio-renal events, at much younger age. It also clearly indicates that smoking increases the risk of deadly consequences in patients with renovascular hypertension and indicate a need for cessation counseling for patients that currently smoke.
